# Growth of Malignant Non-CNS Tumors Alters Brain Metabolome

**DOI:** 10.3389/fgene.2018.00041

**Published:** 2018-02-20

**Authors:** Anna Kovalchuk, Lilit Nersisyan, Rupasri Mandal, David Wishart, Maria Mancini, David Sidransky, Bryan Kolb, Olga Kovalchuk

**Affiliations:** ^1^Department of Neuroscience, University of Lethbridge, Lethbridge, AB, Canada; ^2^Leaders in Medicine Program, Cumming School of Medicine, University of Calgary, Calgary, AB, Canada; ^3^Group of Bioinformatics, Institute of Molecular Biology, National Academy of Sciences, Yerevan, Armenia; ^4^The Metabolomics Innovation Center, Department of Biological Sciences, University of Alberta, Edmonton, AB, Canada; ^5^Department of Oncology, Champions Oncology, Baltimore, MD, United States; ^6^Department of Otolaryngology and Oncology, Johns Hopkins University, Baltimore, MD, United States; ^7^Department of Biological Sciences, University of Lethbridge, Lethbridge, AB, Canada

**Keywords:** tumor brain, non-CNS tumors, metabolomics/metabolite profiling, animal models, brain aging

## Abstract

Cancer survivors experience numerous treatment side effects that negatively affect their quality of life. Cognitive side effects are especially insidious, as they affect memory, cognition, and learning. Neurocognitive deficits occur prior to cancer treatment, arising even before cancer diagnosis, and we refer to them as “tumor brain.” Metabolomics is a new area of research that focuses on metabolome profiles and provides important mechanistic insights into various human diseases, including cancer, neurodegenerative diseases, and aging. Many neurological diseases and conditions affect metabolic processes in the brain. However, the tumor brain metabolome has never been analyzed. In our study we used direct flow injection/mass spectrometry (DI-MS) analysis to establish the effects of the growth of lung cancer, pancreatic cancer, and sarcoma on the brain metabolome of TumorGraft™ mice. We found that the growth of malignant non-CNS tumors impacted metabolic processes in the brain, affecting protein biosynthesis, and amino acid and sphingolipid metabolism. The observed metabolic changes were similar to those reported for neurodegenerative diseases and brain aging, and may have potential mechanistic value for future analysis of the tumor brain phenomenon.

## Introduction

Recent successes in the development of cancer treatments have changed cancer from being a deadly disease to a chronic condition, thereby bringing cancer survivorship and quality of life to the forefront of healthcare. Cancer survivors suffer numerous side effects from treatments, including fatigue and gastrointestinal, hematological, and skin issues. Moreover, they experience chemotherapy-associated cognitive changes spanning across various domains such, as working memory, cognition, executive function, and processing speed. These chemotherapy-induced cognitive changes result in “chemo brain” and affect up to 75% of patients, persisting for years or even decades in one-third of individuals (Janelsins et al., 2011; Ahles, 2012; Andreotti et al., 2015).

Several studies conducted over the past decade have indicated that cognitive impairment occurs long before cancer treatment begins and even before cancer diagnosis (Hurria et al., 2007; Ahles, 2012). These findings suggest that cancer alone (independent of any therapy or treatment) exerts a negative impact on the central nervous system (CNS) (Hurria et al., 2007). However, the mechanisms of cancer-induced cognitive impairment, or “tumor brain,” still need further study.

In our recent studies, we established that the growth of malignant non-CNS tumors resulted in noticeable changes to global gene expression patterns, affecting numerous signaling and metabolic pathways. These alterations in gene expression patterns may in turn impact cellular metabolite levels (Brink-Jensen et al., 2013).

The cellular metabolome is comprised of all the low molecular weight molecules, called metabolites, which are the end products of biochemical and gene expression pathways in cells and tissues. Metabolomics is a relatively new area of research and seeks to analyze metabolome profiles and provide biologically relevant insights into metabolic processes. This is valuable for the analysis of various human diseases, including cancer, neurodegenerative diseases, and aging, yielding both mechanistic insights and new disease biomarkers (Armitage and Barbas, 2014; Jones and C. A. B. International, 2014; Jové et al., 2014; Botas et al., 2015; Ivanisevic and Siuzdak, 2015; Shao et al., 2016; Wishart et al., 2016; Zhang T. et al., 2016; Zhang X. et al., 2016). Metabolomics facilitates the understanding of an organism's physiology and its responses to nutrition and various environmental conditions and stimuli. It has also been proposed as a new tool for exposure studies (Wild, 2005; Lenox, 2015; National Academies of Sciences, 2016; Cheung et al., 2017; Golla et al., 2017). Cancer significantly alters the metabolic profiles the blood, urine and saliva (Armitage and Barbas, 2014; Falegan et al., 2015; Mal, 2016; Shao et al., 2016, 2017; Wishart et al., 2016; Zhang X. et al., 2016; Zhou et al., 2017). However, the tumor brain metabolome has never been analyzed.

In our study, we applied a direct flow injection/mass spectrometry (DI-MS) analysis to determine whether the growth of malignant stage IV non-small cell lung carcinoma (NSCLC), pancreatic cancer, and sarcoma caused alterations in the brain metabolome of TumorGraft™ mice. We discovered that the growth of malignant non-CNS tumors affected metabolic processes in the brain and identified the metabolic fingerprints for tumor brain. The observed metabolic changes were similar to those reported for neurodegenerative diseases and brain aging, and may have significant mechanistic and diagnostic value for future tumor brain research.

## Results and discussion

**Figure 1:**
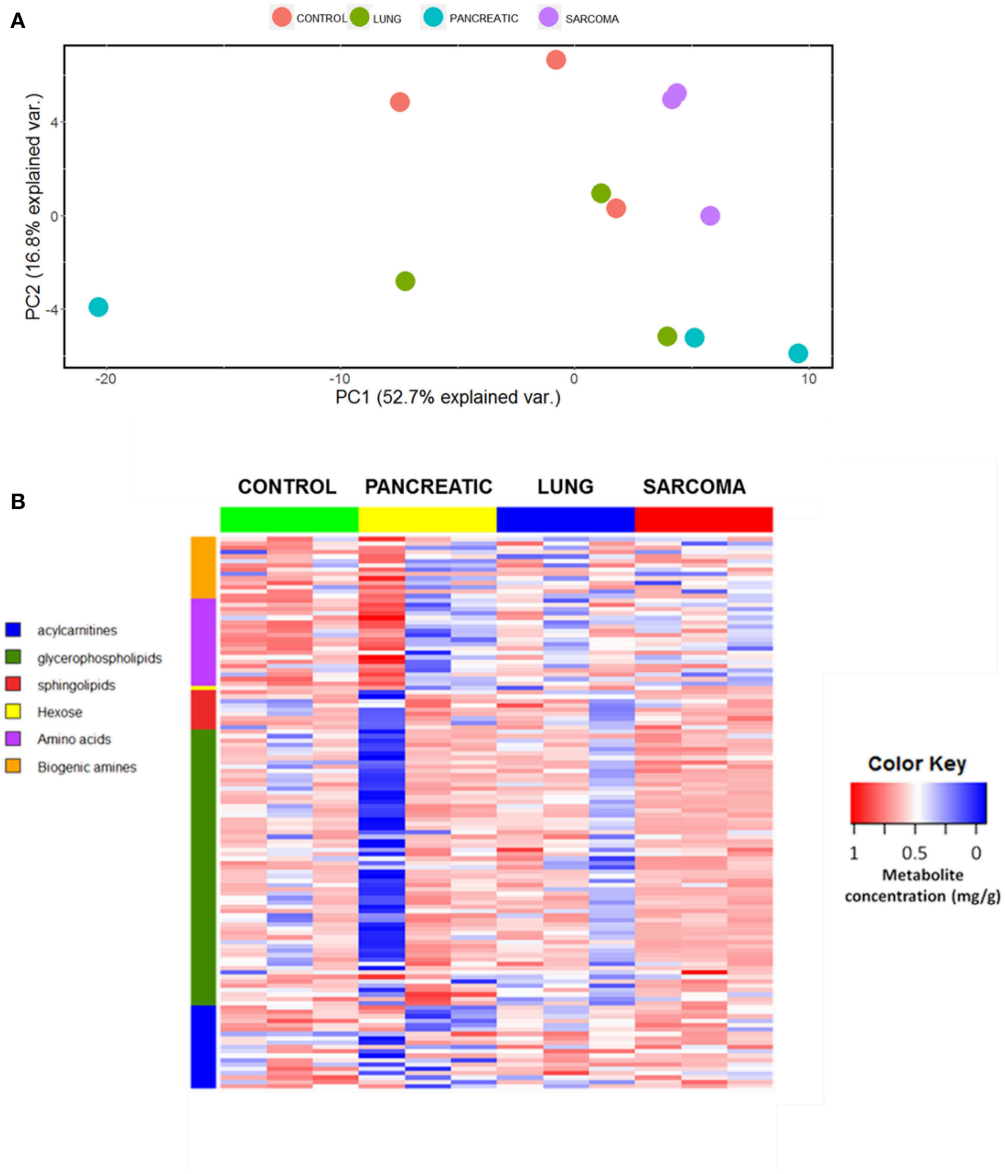
Effects of non-CNS tumor growth on the brain metabolite profiles. **(A)**. PCA plot for the first and the second principal components based on the analysis of all metabolites. **(B)** Metabolite profile heatmap of brain tissues of pancreatic cancer-, lung cancer-, and sarcoma-bearing mice, as compared to controls. Each line represents an individual sample, with 3 samples per group. The heatmap visually represents a metabolic signature of each individual sample, as well as reveals either the up- or down-regulation of metabolites in samples belonging to various groups. The X axis shows sample group; the Y axis depicts individual metabolites in metabolic groups.

We analyzed the levels of various metabolites belonging to acylcarnitines, glycerophospholipids, sphingolipids, hexose, amino acids, and biogenic amines in the brains of lung cancer-, pancreatic cancer-, and sarcoma-bearing tumor graft mice. We identified the metabolic changes and established the metabolic profiles of the brains of tumor-bearing mice. Initially we used the principle component analysis (PCA), a statistical tool to help analyze the sample differences and ascertain the main variables within a multidimensional data set. PCA was based on all the analyzed metabolites. Although there was no distinct clustering observed by the first and second principal components, the control group clearly separated from the samples of pancreatic tumor-bearing mice across the second component (Figure 1A). Likewise, the heatmap of various analyzed metabolites revealed high intra-sample variability in metabolic profiles for all the groups (Figure 1B and Figure S1). Nonetheless, it showed that non-CNS tumor growth led to changes in the metabolic activity in the brain of tumor-bearing animals.

**Figure 2:**
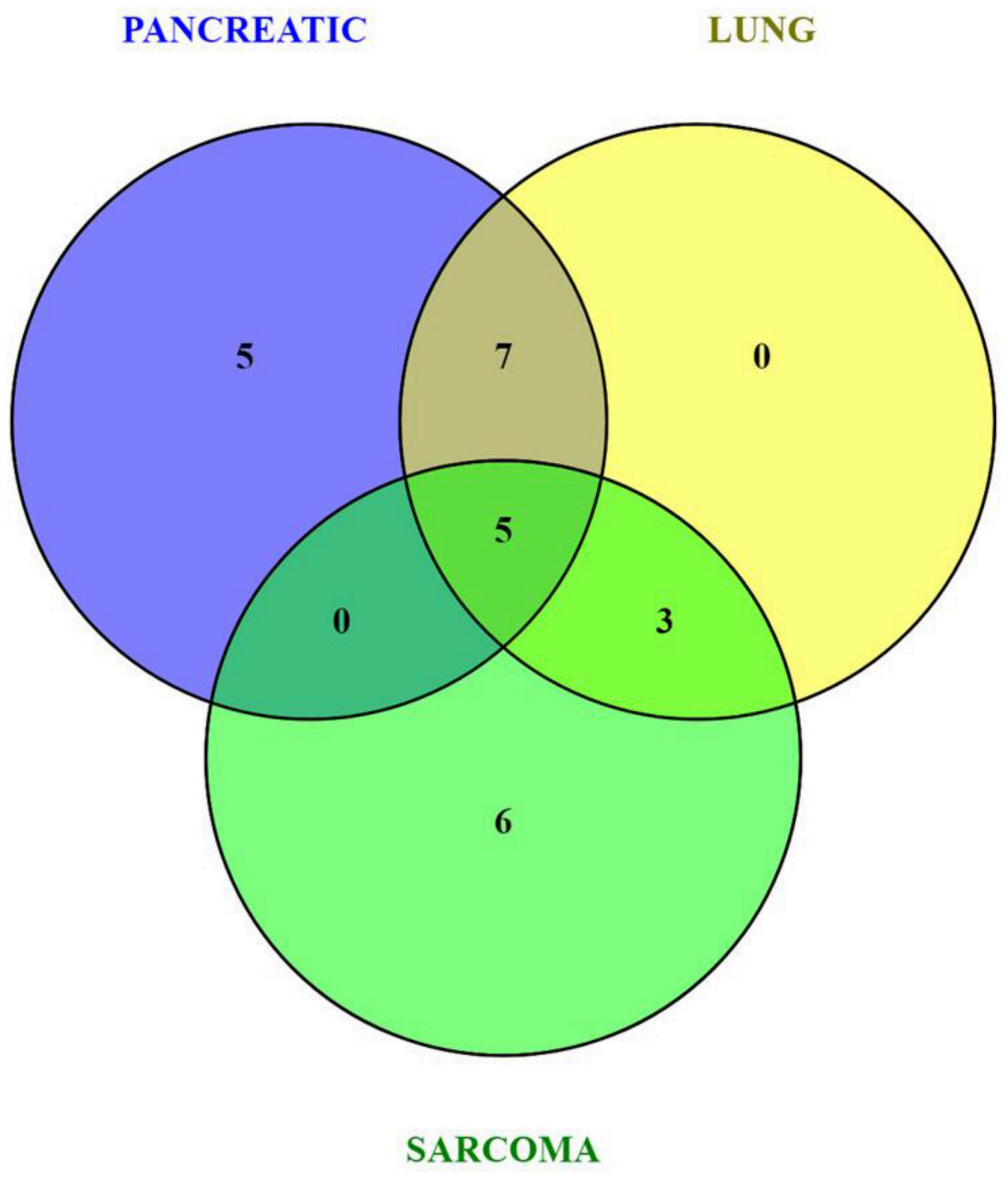
Venn diagram of metabolic pathways altered in the brains of pancreatic cancer-, lung cancer-, and sarcoma-bearing mice, as compared to controls.

**Figure 3:**
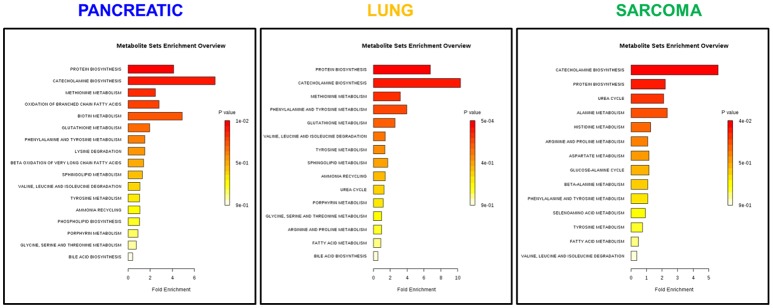
Analysis of metabolic pathways bi-directionally altered in the brains of pancreatic cancer-, lung cancer-, and sarcoma-bearing mice, as compared to controls. Metabolite set enrichment analysis allowed to establish which pathways were affected in the brains of tumor-bearing mice as compared to controls. Plots show the results of overrepresentation analysis of various metabolic pathways based on individual metabolites that were identified in each sample within experimental groups. Only one pathway “protein [amino acid] biosynthesis” was consistently up-regulated in all three cancers. Color coding represents the p values for metabolic pathways (dark red showing the least p value and white the highest *p*-value). The lengths of the bars represent the fold enrichment. For pancreatic cancer, the Holm-Bonferroni adjusted p value range was from 0.01 (dark red) to 0.9 (white). For lung cancer, *p*-values ranged from 0.0005 (dark red) to 0.9 (white). For sarcoma, p values ranged from 0.04 to 0.9.

**Figure 4:**
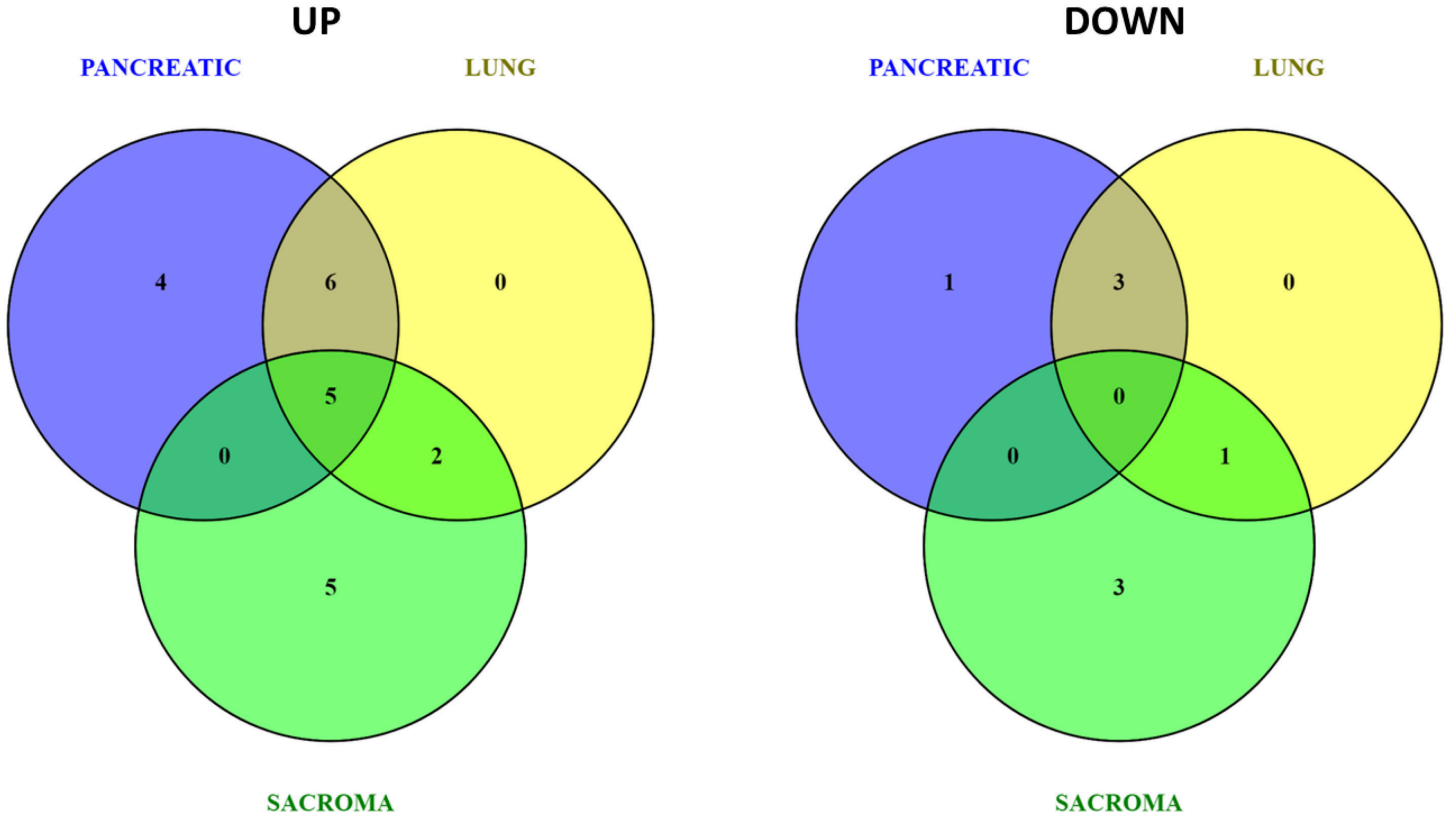
Venn diagrams of up- and down-regulated metabolic pathways in the brains of pancreatic cancer-, lung cancer-, and sarcoma-bearing mice, as compared to controls.

**Figure 5:**
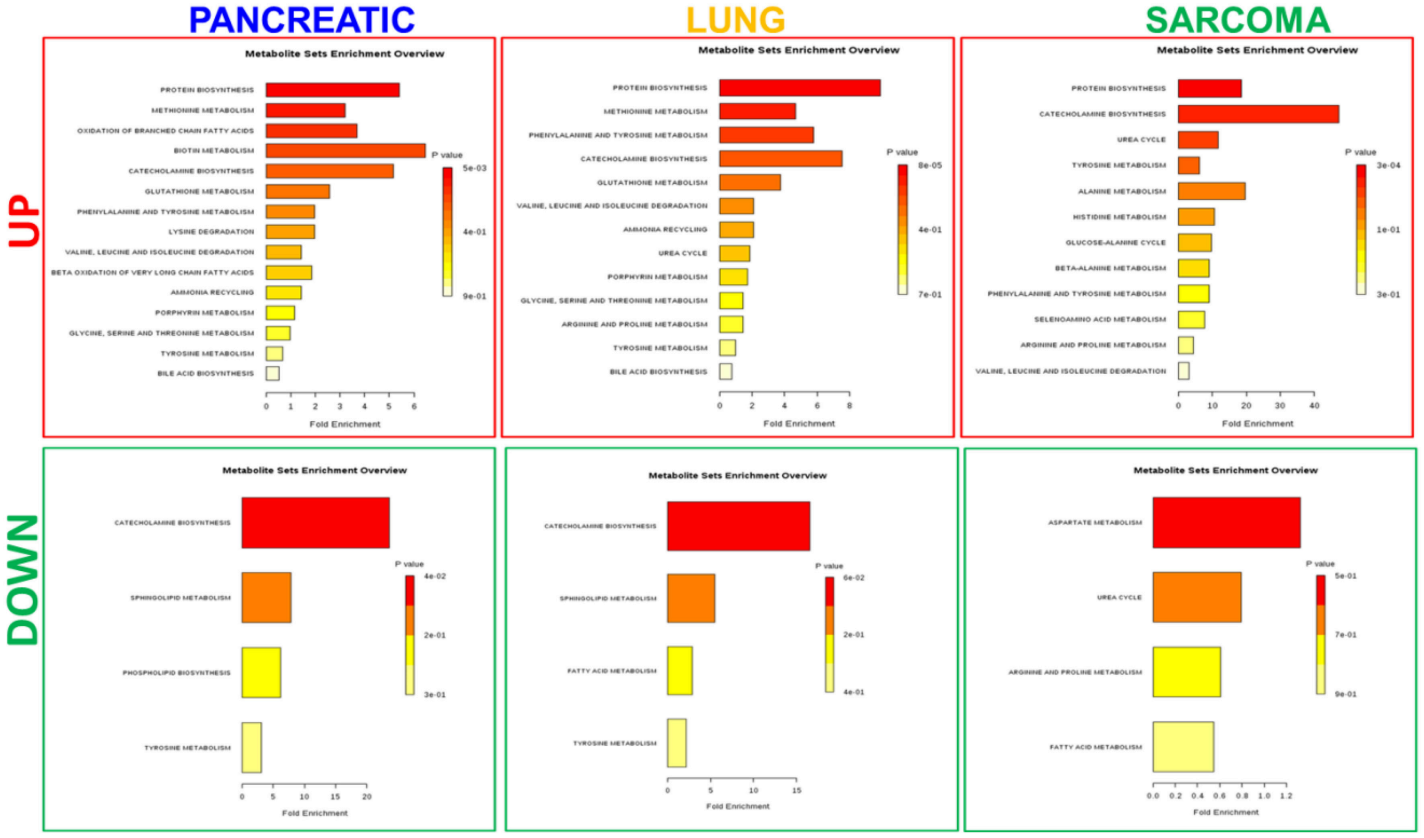
Analysis of metabolic pathways up- and down-regulated in the brains of pancreatic cancer-, lung cancer-, and sarcoma-bearing mice, as compared to controls. Metabolite set overrepresentation analysis allowed to establish which pathways were affected in the brains of tumor-bearing mice as compared to controls. Two separate lists of up- and down-regulated metabolites were supplied for the analysis (indicated in the left part of the figure as “UP” or “DOWN”). Color coding represents the p values for metabolic pathways (dark red showing the least p value and white the highest *p*-value). The lengths of the bars represent the fold enrichment.

Because of small sample sizes, we haven't observed statistical significance in metabolite changes. However, metabolites exist and function as part of complex metabolic pathways and networks; thus, focusing on individual, or even groups of, metabolites is not informative. To gain an in-depth understanding of the magnitude and functional significance of the observed changes, we analyzed the metabolites in the context of metabolic pathways and performed the metabolite set enrichment analysis (MSEA) using MetaboAnalyst software (Xia et al., 2015; Xia and Wishart, 2016). MSEA allowed us to establish which pathways were affected in the brains of tumor-bearing mice as compared to controls. We also compared the metabolic pathway patterns between the groups. The analysis revealed several interesting patterns: 17 pathways were deregulated in the brains of the pancreatic cancer-bearing mice, 15 in the brains of the lung cancer-bearing mice, and 14 in the brains of the sarcoma-bearing animals (Figures 2, 3). Five pathways were affected in all groups. To understand if the observed enrichment patterns were caused by up- or down- regulated metabolites, we also performed enrichment analysis on up- and down-regulated metabolites separately. Some pathways were identified only in the lists of upregulated, some—only in the lists of downregulated metabolites, while several exhibited bi-directional alterations in the pathway metabolites (Figures 4, 5).

**Figure 6:**
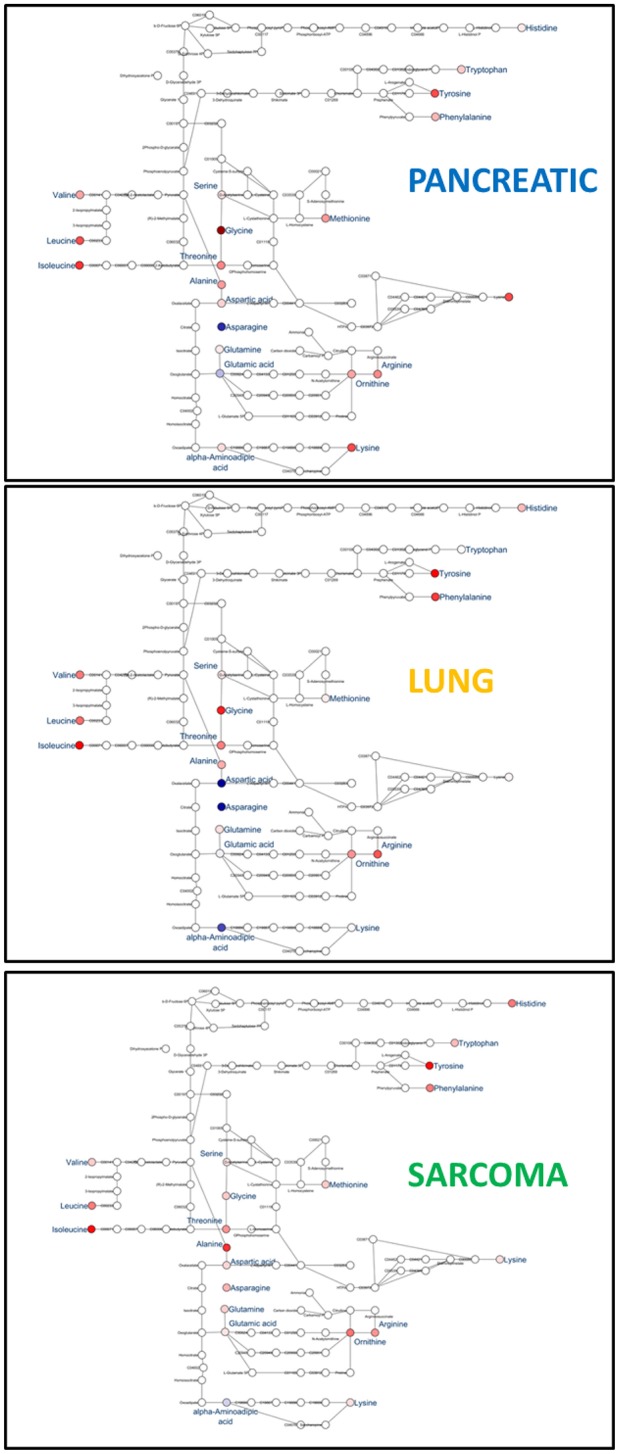
Schematic representation of KEGG Biosynthesis of amino acids pathway in the brains of pancreatic cancer-, lung cancer-, and sarcoma- bearing mice, as compared to controls. Red denotes upregulation as compared to controls; blue denotes downregulation to as compared to controls (see Table 1 for numeric values).

Of the five pathways that were affected in all three experimental groups, the process of protein biosynthesis was consistently upregulated (with Holm *p* < 0.05 in all three cancers). Furthermore, in all groups, amino acid metabolism was affected, as evidenced by the changes in phenylalanine and tyrosine metabolism, as well as valine, leucine, and isoleucine degradation. (Figures 3, 5, 6). The fold changes of all the amino acids as compared to controls are represented in Table 1. In the brain, excess amino acids are usually used for energy production; and oftentimes, when neurons cannot catabolize glucose, they oxidize amino acids as alternative energy sources.

**Table 1:** Levels of amino acids in the brains of tumor-bearing animals (fold changes as compared to control animals).

**Compound**	**Pancreatic**	**NSCLC**	**Sarcoma**
Alanine	1.36	1.30	1.75
Isoleucine	1.75	1.93	1.89
Leucine	1.64	1.53	1.48
Lysine	1.66	1.03	1.12
Methionine	1.37	1.07	1.19
Ornithine	1.34	1.40	1.51
Phenylalanine	1.27	1.72	1.48
Proline	0.89	0.98	1.13
Serine	1.12	1.10	1.10
Threonine	1.45	1.47	1.39
Tryptophan	1.20	1.02	1.25
Arginine	1.43	1.62	1.39
Tyrosine	1.66	1.93	1.89
Asparagine	0.86	0.83	1.28
Aspartic acid	1.17	0.83	1.15
Citruline	1.15	1.08	0.66
Glutamine	1.08	1.12	1.17
Glutamic acid	0.95	0.99	1.10
Glycine	2.47	1.82	1.20
Histidine	1.08	1.25	1.49

*Red denotes upregulation; blue denotes downregulation, albeit the observed changes were not statistically significant after multiple test corrections*.

These results suggest that the tumor–brain phenomenon may be similar to neurodegeneration and aging (Griffin and Bradshaw, 2017). Several previous studies have shown altered amino acid levels in the brains of Alzheimer's disease (AD) patients and AD mouse models, even though the functional and mechanistic significance of these changes has not yet been established (Griffin and Bradshaw, 2017). The changes in these AD studies were similar to the ones noted in tumor brain.

Decreases in the levels of amino acid in the brain, or the deregulation of the machinery that metabolizes them, may cause neuronal death. Likewise, amino acid oxidation, and catabolism that leads to the release of ammonia may also cause neuronal apoptosis. This is due to much lower levels of several urea cycle enzymes that are needed for ammonia detoxification in neurons and glia. One of these enzymes is glutamine synthetase, which sequesters ammonia into glutamine and is expressed at very low levels in neurons. Changes in protein synthesis may cause neuronal cell death and thereby contribute to neurodegeneration. Altered amino acid metabolism was previously linked to neurological deficits in dementia patients (Liu et al., 2014; Griffin and Bradshaw, 2017), and aromatic amino acids (phenylalanine and tryptophan) increased in the AD brain (Xu et al., 2016). Furthermore, alterations in protein biosynthesis pathways were previously reported in the brains of transgenerationally stressed animals (Kiss et al., 2016), and were suggested to be related to neurological deficits.

**Figure 7:**
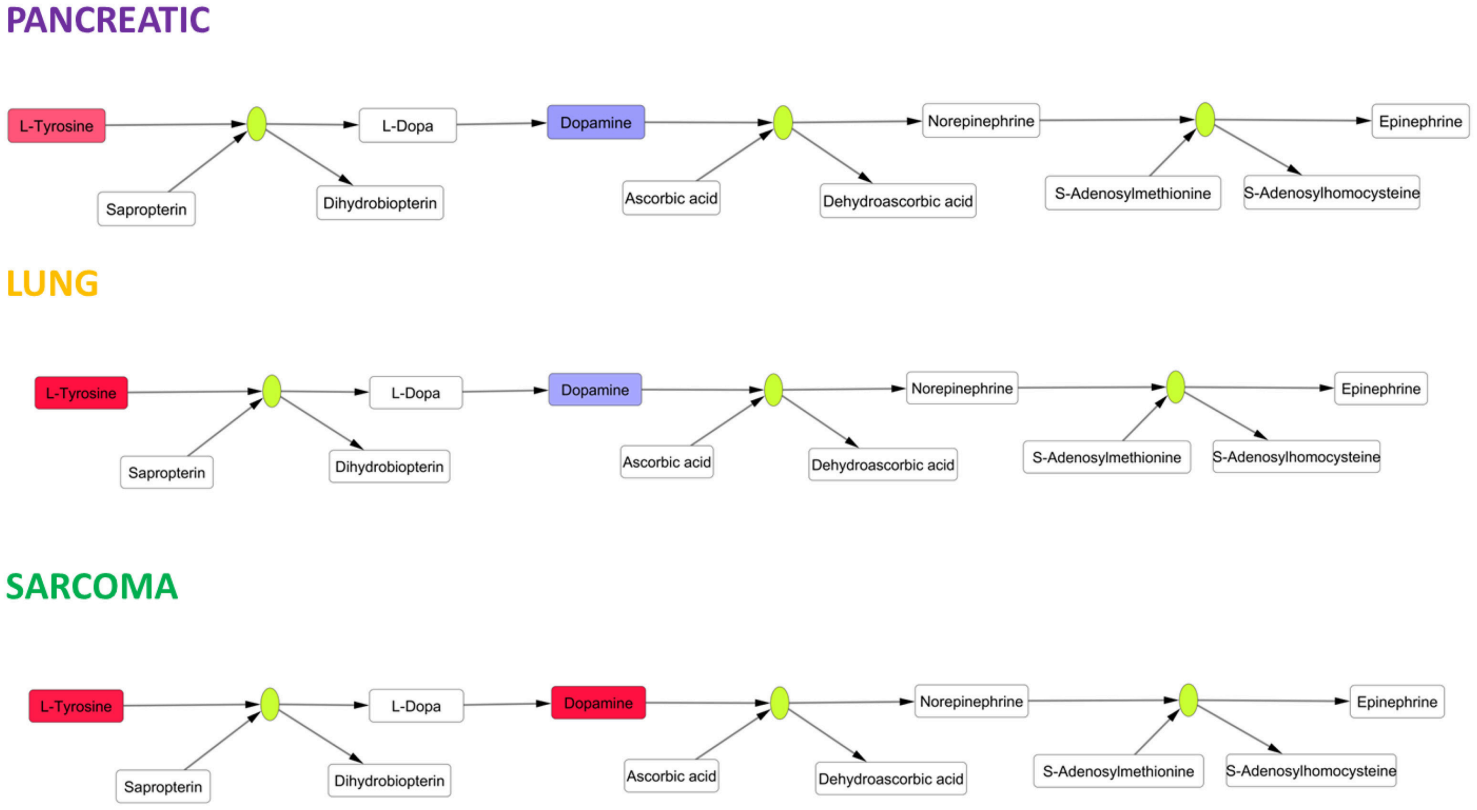
Schematic representation of SMPDB Catecholamine biosynthesis pathway. Two compounds belonging to this pathway were measured: L-Tyrosine and dopamine. L-Tyrosine was up-regulated in all three cancer-bearing groups, while dopamine was only up-regulated in sarcoma-bearing groups. Red denotes upregulation as compared to controls; blue denotes downregulation as compared to controls. Green nodes represent enzymes.

Amino acids play pivotal roles in neural cells as neurotransmitters and their precursors. We observed small increases in the levels of glutamate, an excitatory neurotransmitter and precursor of inhibitory neurotransmitter gamma-aminobutyric acid (GABA) (Xu et al., 2016). Glutamate is involved in the pathophysiology of Alzheimer's disease, and altered glutamate levels were previously reported in AD patients (Xu et al., 2016). Aromatic amino acids are precursors of cerebral neurotransmitters, monoamine (serotonin) and catecholamine (dopamine, norepinephrine and epinephrine) (Xu et al., 2016). We observed significant changes (according to Holm-Bonferroni *p*-values) in catecholamine metabolism in the brain tissues of pancreatic cancer-, lung cancer-, and sarcoma-bearing mice (Figure 7). Catecholamine biosynthesis had differential regulation due to dopamine down-regulation in pancreatic and lung cancer-bearing mice and to dopamine up-regulation in sarcoma-bearing animals.

The observed alterations in catecholamine biosynthesis and the increases in levels of aromatic amino acids (phenylalanine and tryptophan) may cause neurotransmitter imbalances analogous to those previously exemplified by lowered levels of serotonin, dopamine, and norepinephrine in the AD brain (Storga et al., 1996; Matthews et al., 2002; Garcia-Alloza et al., 2005; Xu et al., 2016), again suggesting a potential link between tumor–brain and neurodegeneration. The causes of protein and amino acid metabolism deregulation need to be further analyzed. Altered protein biosynthesis may occur as part of a compensatory or repair mechanism in response to oxidative stress and oxidative DNA damage. In addition, more studies are needed to further analyze the roles of deregulated protein and amino acid metabolisms in tumor brain and the mechanisms leading to this deregulation.

Along with protein and amino acid metabolism and amino acid degradation, we observed changes in the urea cycle of the brains of the tumor-bearing mice. The urea cycle was enriched in the brains of the lung cancer- and sarcoma-bearing animals, but not in the pancreatic cancer-bearing animals. One of the constitutive compounds of the urea cycle—citrulline—which is also a member of the amino acid pathway, was downregulated in the brains of the sarcoma animals, but upregulated in the brains of the lung and pancreatic cancer animals (Table 2). This deregulation may be the consequence of altered protein metabolism, and its roles remain elusive; however, it is yet another pathway implicated in neurodegeneration (Xu et al., 2016). We also noted changes in methionine metabolism in the brains of all experimental groups (Table 3). Previously, altered methionine metabolism was reported to occur upon traumatic brain injury (Dash et al., 2016).

**Table 2:** Levels of urea cycle components in the brains of tumor-bearing animals (fold changes as compared to control animals).

**Compound name**	**Pancreatic**	**Lung**	**Sarcoma**
Citrulline	1.15	1.08	0.66
L-Glutamic acid	0.95	0.99	1.10
L-Aspartic acid	1.17	0.83	1.15
L-Glutamine	1.08	1.12	1.17
L-Arginine	1.43	1.62	1.39
Ornithine	1.34	1.40	1.51
L-Alanine	1.36	1.30	1.75

*Red denotes upregulation; blue denotes downregulation, albeit the observed changes were not statistically significant after multiple test corrections*.

**Table 3:** Methionine metabolism (fold changes as compared to control animals).

**Compound name**	**Pancreatic**	**Lung**	**Sarcoma**
Putrescine	0.97	2.14	0.93
L-Serine	1.12	1.10	1.10
L-Methionine	1.37	1.07	1.19
Glycine	2.47	1.82	1.20
Spermidine	1.76	1.60	1.42

*Red denotes upregulation; blue denotes downregulation, albeit the observed changes were not statistically significant after multiple test corrections*.

This study is the first to analyse and report the effects of non-CNS tumor growth on the brain metabolome. Gene expression pathways control metabolic pathways and, as such, the cellular metabolome constitutes the outcome of global gene expression. Overall, we observed altered regulation of protein synthesis, amino acid metabolism and degradation, sphingolipid metabolism, and several other metabolic pathways in the brains of tumor-bearing animals. Metabolic changes, as well as the vast majority of other molecular effects observed in this study, were previously implicated in aging and age-related neurodegenerative diseases such as AD and dementia. These changes may be associated with neurodegeneration and aging and thus may have implications for cancer patients as they age.

One way to better understand the functional significance and interconnection between mechanisms and pathways in tumor brain, is through integrating multi-omics data. Such a large amount of data could only be handled using special approaches—e.g., sophisticated machine learning techniques—that have shown greater promise in handling large amounts of complex, nonlinear, and multidimensional datasets than traditional approaches. Machine learning, and deep learning in particular, provides a tremendous opportunity to identify novel biomarkers of diseases and conditions and establish mechanisms of diseases and treatment responses (Mamoshina et al., 2016; Putin et al., 2016; Borisov et al., 2018).

Hence, in the future it would be important to apply machine learning and correlate metabolome levels with the levels of gene expression, as well as with epigenome alterations. Multi-level integration of various molecular domains may shed light on the molecular mechanisms and outcomes of tumor brain. It may also help develop tumor brain diagnostic and prognostic biomarkers, and guide the development of appropriate mitigation and prevention strategies. Moreover, further studies are needed to compare metabolome profiles of tumors, the brain, as well as blood in context of tumor brain models, as well as to establish the effects of chemotherapy on brain metabolome.

Future studies are needed to further dissect organismal repercussions of the observed changes, those, indeed may constitute adaptive changes or deleterious ones. It would be also important to further correlate metabolomic changes with cognitive and behavioral outcomes in tumor brain.

## Materials and methods

### Animal model

To study the effects of non-CNS tumor growth on the brain metabolome, we used the mouse TumorGraft models developed and provided by the precision medicine company Champions Oncology, Inc. (Baltimore, MD). We obtained frozen brain tissues of TumorGraft mice carrying pancreatic cancer, sarcoma and lung (NSCLC) cancer patient-derived xenografts (PDX). Patients diagnosed with sarcoma, pancreatic, and lung cancer had their tumors surgically removed and small pieces of the tumor were implanted in mice. This allowed the production of personalized TumorGraft mouse models for the development of precision oncology strategies. All patients gave their full informed consent for the use of their tumor tissues for research purposes.

The animal experiments were approved by Institutional Animal Care and Use Committee protocols. To generate mouse TumorGrafts, small tumor tissue fragments with both malignant cells and supportive stroma were implanted into the flanks of 6-week-old immunodeficient female mice (female *nu*/*nu* athymic mice; Harlan Laboratories, Indianapolis, IND) and propagated as previously described(Bertotti et al., 2011; DeRose et al., 2011; Hidalgo et al., 2011; Morelli et al., 2012; Stebbing et al., 2014). When the TumorGrafts reached more than 200 mm^3^, the animals were divided into groups of three. Tumor growth was monitored; tumor dimensions were regularly measured and tumor volumes were calculated as previously described (Stebbing et al., 2014). Intact animals (no tumor, no treatment, *n* = 3) served as baseline controls. Animals were euthanized by Euthansol overdose. The brains of the animals were removed from their skulls and split in half. They were then frozen in liquid nitrogen and stored at −80°C until further metabolomics analysis.

### Tissue sample extraction

Metabolomic profiling was carried out at The Metabolomics Innovation Center, Edmonton, AB using mouse left hemibrains. Each tissue sample was weighed and its mass was recorded, and a tissue exaction buffer was prepared [85 mL MeOH + 15 mL phosphate buffer solution (10 mM)]. Next, each tissue sample was homogenized in the tissue extraction buffer at a volume three times that of the tissue. For example, 90 uL of tissue extraction buffer was used for 30 mg of tissue. Then, the homogenized samples were centrifuged at 14,000 rpm and the supernatant was transferred into a new vial. The resultant supernatant was stored at −20°C until further analysis by liquid chromatography tandem-mass spectrometry (LC-MS/MS).

### Direct flow injection mass spectrometric compound identification and quantification

We applied a targeted quantitative metabolomics approach to analyze the samples by using direct flow injection mass spectrometry (AbsoluteIDQ™ Kit). This kit assay, in combination with a 4000 QTrap (Applied Biosystems/MDS Sciex) mass spectrometer, was used for the targeted identification and quantification of a large number of endogenous metabolites, including amino acids, acylcarnitines, glycerophospholipids, sphingolipids, and sugars. This method combines the derivatization and extraction of analytes with selective mass-spectrometric detection using multiple reaction monitoring (MRM) pairs. Isotope-labeled internal standards are integrated in a Kit plate filter for metabolite quantification.

The AbsoluteIDQ™ kit contains a 96-deep well plate with a filter plate attached by sealing tape, as well as reagents and solvents used to prepare the plate assay. Fourteen wells in the kit were used for the following: one blank, three zero samples, seven standards, and three quality control samples that were provided with each kit. The samples were left to thaw on ice. Once thawed, they were vortexed and then centrifuged at 13,000 × g. Next, 10 μL of supernatant for each sample were loaded on a filter paper of the kit plate and dried in a stream of nitrogen. Next, 20 μL of a 5% solution of phenyl-isothiocyanate was added for derivatization. After incubation, the filter spots were dried again using an evaporator. Extraction of the metabolites was then achieved by adding 300 μL of methanol containing 5 mM ammonium acetate. The extracts were obtained by centrifugation in the lower 96-deep well plate, followed by a dilution step with a kit MS running solvent. A mass spectrometric analysis was performed on an API4000 Qtrap® tandem mass spectrometry instrument (Applied Biosystems/MDS Analytical Technologies, Foster City, CA) equipped with a solvent delivery system. The samples were delivered to the mass spectrometer by liquid chromatography, followed by a direct injection (DI) method. Biocrates MetIQ software was used to control the entire assay workflow, from sample registration and the automated calculation of metabolite concentrations and to the exporting of the data into other data analysis programs. A targeted profiling scheme was used to screen for known small molecule metabolites using multiple reaction monitoring, neutral loss, and precursor ion scans.

### In-depth analysis of brain metabolome

For each cancer-bearing animal sample, we obtained the mean metabolite level value and divided it by that of the control samples to get the fold change (FC) values. Then, we submitted the up- regulated genes (FC > 1.5) or down-regulated genes (FC < 0.8), or both, to MetaboAnalyst to perform Metabolite set enrichment analysis (MSEA) (Xia et al., 2012, 2015; Xia and Wishart, 2016). The principal component analysis (PCA) (Raychaudhuri et al., 2000) and hierarchical clustering were performed in R, with the “stats” package. Pathway analysis was performed in Cytoscape, based on pathway diagrams obtained from KEGG (http://www.kegg.jp/kegg/pathway.html) and SMPDB (http://smpdb.ca/) pathway databases.

## Author contributions

AK planned and designed the study, conducted initial samples processing, data analysis, paper preparation and revisions; LN conducted bioinformatic analysis; RM from the metabolomics platform run the samples; DW is a director of the metabolomics platform; MM and DS provided animal samples; BK and OK planned and designed the study, supervised it, revised the manuscript. All authors read the manuscript.

### Conflict of interest statement

The authors declare that the research was conducted in the absence of any commercial or financial relationships that could be construed as a potential conflict of interest.

## Supplementary material

Figure S1 Metabolite profile clustered heatmap of brain tissues of pancreatic cancer-, lung cancer-, and sarcoma-bearing mice, as compared to controls.
