# Supplementary figures and images for: Growth of Malignant Non-CNS Tumors Alters Brain Metabolome
Source: Front Genet. 2018 Feb 20;9:41. doi: 10.3389/fgene.2018.00041 (PMC5826252; doi:10.3389/fgene.2018.00041)

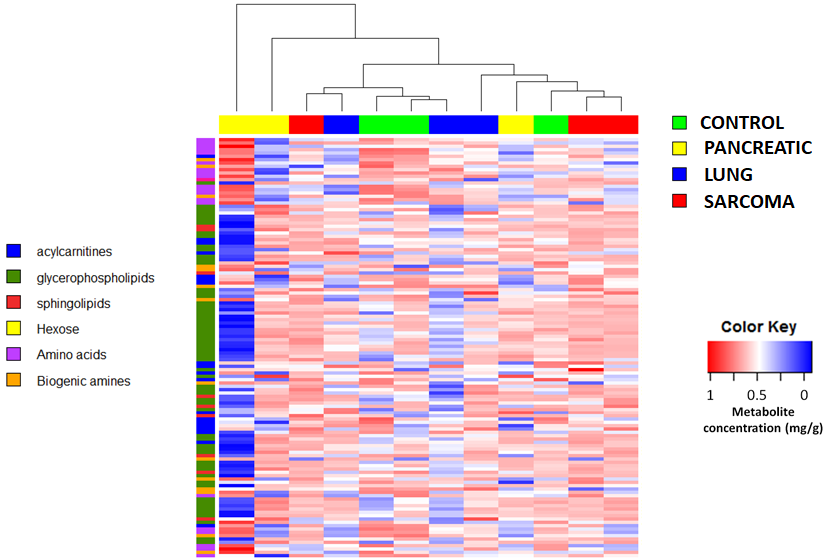

Supplement: Figure S1 — Metabolite profile clustered heatmap of brain tissues of pancreatic cancer-, lung cancer-, and sarcoma-bearing mice, as compared to controls. [file Image1.PNG]
